# Does the Colonizing Population Exhibit a Reduced Genetic Diversity and Allele Surfing? A Case Study of the Midday Gerbil (*Meriones meridianus* Pallas) Expanding Its Range

**DOI:** 10.3390/ani14182720

**Published:** 2024-09-20

**Authors:** Olga N. Batova, Nikolay I. Markov, Sergey V. Titov, Andrey V. Tchabovsky

**Affiliations:** 1 Laboratory for Population Ecology, Severtsov Institute of Ecology and Evolution, Russian Academy of Sciences, 33 Leninskii Pr., 119071 Moscow, Russia; batova_olga@mail.ru (O.N.B.); nimarkov@ipae.uran.ru (N.I.M.); 2 Laboratory for Game Animals Ecology, Institute of Plant and Animal Ecology, Ural Branch of Russian Academy of Sciences, 202a 8 Marta St., 620142 Ekaterinburg, Russia; 3 Department of Zoology and Ecology, Penza State University, 40 Krasnaya St., 440026 Penza, Russia; svtitov@yandex.ru

**Keywords:** range expansion, colonization, desertification, rangelands, genetic structure, rodents

## Abstract

**Simple Summary:**

We live in a changing world, and human-induced landscape changes cause shifts and expansions of ranges of living organisms worldwide. During range expansions, animals and plants invade and colonize new areas, which may have dramatic ecological and evolutionary consequences. We studied the genetic consequences of range expansion in the desert rodent, currently colonizing new areas in Kalmykia (Southern Russia) following the desertification of rangelands. We found that genetic diversity in the colonizing population was lower than in the range core. Moreover, the colonizing population exhibited strong spatial structuration and increased frequencies of genetic variants (alleles) rare in the source population—signatures of allele surfing, a phenomenon theoretically predicted but, so far, rarely observed in natural populations. Our findings provide new insights into understanding the genetic consequences of range expansions and are important for managing expanding populations and invasions.

**Abstract:**

Colonizing populations at the leading edge of range expansion are expected to have a reduced genetic diversity and strong genetic structure caused by genetic drift and allele surfing. Until now, few studies have found the genetic signatures of allele surfing in expanding wild populations. Using mtDNA markers, we studied the genetic structure of the population of midday gerbils (*Meriones meridianus*) expanding their range to the west in Kalmykia (southern Russia) following the new cycle of desertification, re-colonizing areas abandoned in the mid-2010s. In the colonizing population, we found a reduced genetic diversity, the redistribution of haplotype frequencies—in particular, in favor of variants rare in the core population—and strong genetic structure combined with strong differentiation from the core population—patterns suggestive of allele surfing on the wave of expansion. In terms of genetic diversity and spatial structuration, the western edge population sampled in 2008 before its collapse in 2017 occupies the intermediate position between the current colonizing and core population. This suggests that reduced genetic diversity and increased genetic differentiation are general features of marginal populations, enhanced by the founder and allele-surfing effects at the leading edges of expanding ranges.

## 1. Introduction

Invasive species or species expanding their ranges and colonizing new areas offer unprecedented opportunities to study rapid evolutionary changes [1]. Colonizing populations are expected to be genetically different from the source population [2]. Moreover, evidence is accumulating that genetic changes are not only common during colonization but also may contribute directly to colonization success [3,4,5,6,7]. The differences in genetic structure between the source and colonizing populations can arise from (i) the founder effect, which is supposed to decrease genetic diversity in populations of colonists [7], and (ii) allele surfing, i.e., a process that increases the frequencies of neutral, deleterious, or advantageous mutations, rare in the source population, by their “surfing” on the wave of expansion, leading to changes in allele frequencies, stronger spatial genetic structure, and spatial sorting of genotypes [8,9,10,11,12,13]. The colonization from multiple sources, propagule pressure (high number of dispersing individuals), and genetic admixture at the leading edge, along with the influx of immigrants from the core population, can compensate for the loss of genetic diversity caused by drift [2,14,15,16].

A relative contribution of these processes in the genetic profile of the colonizing population is specific to each colonization event and depends on many factors. For example, Uller and Leimu [17] reported that mammals and insects show higher loss of genetic diversity during range expansion than other vertebrate and invertebrate taxa, while plants demonstrated much higher genetic diversity in the “introduced” versus “native” populations. Also, for mammals, Schmidt et al. [18] showed the core–edge variation is strongly species-specific, with a general pattern of a reduction in genetic diversity and an increase in the genetic differentiation toward the range edges. Thus, the changes in genetic diversity and the structure of expanding populations may be taxa-specific and depend on the history of colonization. An influential study by Ibrahim et al. [19] shows that genetic differentiation within expanding populations is strongly affected by the model of dispersal (stepping-stone, island, or leptokurtic), which in turn depends on the community dynamics in the areas of expansion. That is why each new case study of colonization genetics is an essential contribution to understanding the factors and mechanisms underlying colonization and predicting its success, as well as its ecological and evolutionary consequences. Such studies are of particular importance when colonization originates not from intentional or nonintentional translocations of organisms but rather reflects changes at the ecosystem level caused by global climate change and/or human-induced landscape transformations.

In this study, we report the case of the midday gerbil (*Meriones meridianus* Pallas 1773) in Kalmykia. The midday gerbil is a small (50–60 g of adult body mass), short-lived (ca. eight-month life expectancy), psammophilous, mainly granivorous rodent that inhabits more or less fixed sandy patches, avoiding closed tall-grass habitats [20,21]. The range of the midday gerbil extends across northeastern Ciscaucasia, the northern Caspian region, central and eastern Kazakhstan, northwestern China, and southwestern Mongolia [22]. In Kalmykia, at the western limit of the species’ range, the dynamics of its abundance and distribution follow the desertification–steppification cycles, which are determined by fluctuations in the grazing impact and climate [20,23,24,25,26,27]. A rare opportunity to observe colonization in real time was provided by the new cycle of desertification caused by increasing livestock production, which launched the expansion of the psammophilous midday gerbil range to the west in 2019, where they re-colonized re-emerging desert habitats abandoned in previous steppe times [28].

We have asked whether the newly established populations in the western part of the range (colonists) are genetically different from the core eastern population that survived the period of population decline and range contraction. We tested the following hypotheses: (i) The genetic diversity in the colonizing populations is reduced compared to the source population due to the founder effect; (ii) alternatively, it is not lower than in the source population, suggesting an influx of immigrants from the core area, colonization from multiple sources, and/or gene admixture; and (iii) the spatial genetic structure of the colonizing population is stronger than in the source population, and the frequencies of alleles rare in the source population are higher in the colonizing population due to allele surfing.

## 2. Materials and Methods

### 2.1. Study Area

**Figure 1:**
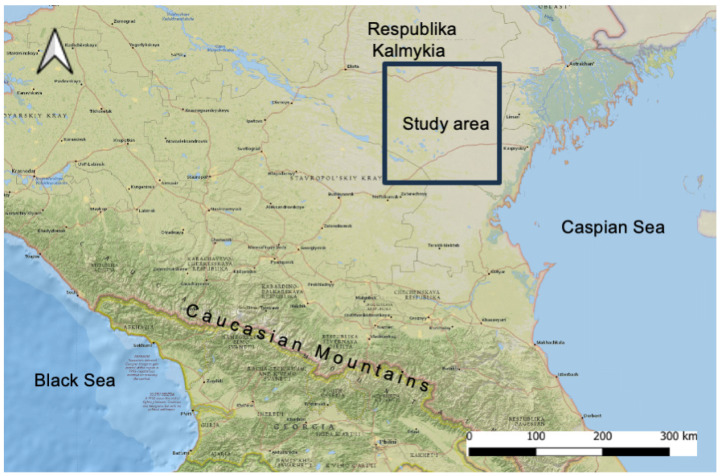
The location of the study area.

The field data were collected in the Southern Kalmykia and the neighboring east Astrakhanskii region (Figure 1). The study area is a semi-desert with vegetation composed of ephemerals, annuals, perennial sod grasses, psammophilous herbage, and subshrubs. Here, gerbils burrow in patchily distributed desert habitats on semi-stabilized hilly sands isolated by a matrix of unsuitable semi-desert loamy plains or tall-grassed stabilized sandy patches. In response to the expansion of steppe vegetation in the 1990s, the population of the midday gerbil sharply declined in the southeastern part of Kalmykia, and the border of its range moved to the east by the mid-2010s [25,26,27]. The new desertification cycle that started in the 2010s has favored the spread of desert habitats and the expansion of the midday gerbil range in the opposite direction—to the west.

**Figure 2:**
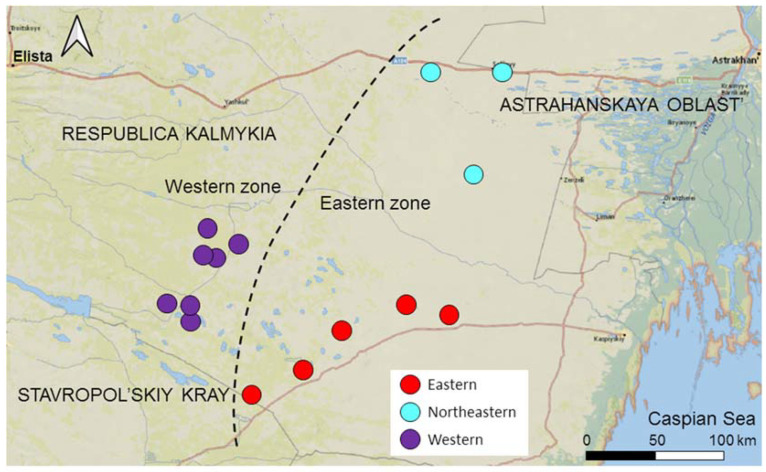
Map of the study area. Subpopulations are shown in different colors. The dashed line separates the Eastern and Western zones.

We divided the entire area into two zones not separated by an ecological or geographical barrier: the Western zone—the area of expansion, and the Eastern zone—the core area (Figure 2). The Western zone extends 50 km from the western border of the range to the border of the Eastern zone. Gerbils have inhabited the Western zone since at least 1980 [24,25]; however, in 2017, the local population collapsed, and gerbils disappeared from the entire Western zone [26]. We continued monitoring the abandoned Western zone, inspecting sandy patches potentially suitable for gerbils for the presence of burrows twice a year (in mid-April–mid-May and mid-September–mid-October) to be ready for the gerbils to come back following the new cycle of desertification [28]. If gerbils settled (in spring or the following autumn) in the patch that had remained vacant since 2017, we considered it a colonizing event. The invasion of gerbils into the Western zone began in the autumn of 2019, with the first few colonists appearing in the two sites at its eastern borders. Since then, new colonies have emerged in the Western zone every year, discovered by regular inspections during spring and autumn sessions [28]. The number of founders of new colonies varied from several to 10–20 gerbils. In three years, the colonizing population rapidly expanded and increased in numbers (see [28] for the population dynamics and demography), reaching probably several hundred individuals. The Eastern zone remained inhabited by gerbils after the population collapse in the west, and gerbils have persisted here since at least the 1960s [23,28]. The Eastern zone extends approximately 100 km from the Western zone to the east and northeast towards the range core. We subdivided the Eastern zone into two sectors, representing two subpopulations based on their geographical location: the Eastern and the Northeastern (Figure 2).

### 2.2. Data Collection

We collected tissue samples (clipped toes) in the Western and Eastern zones, trapping gerbils twice a year: in spring (mid-April–mid-May) and in autumn (mid-September–mid-October). In spring, we trapped animals that survived the winter (adults), and in autumn, both adults and young of the year. We used modified non-commercial Shchipanov live traps [29] and marked gerbils individually with animal ID microchip complaint transponders 1.25 × 7 mm (FOFIA, Wuxi, China). The details on the trapping procedure are available elsewhere [30]. In this study, we used data collected in 2019–2021 in the core population (the Eastern zone) and the colonizing population (the Western zone) comprising the colonists of the first to fourth generation. In addition, we used samples collected in the Western zone in 2008, i.e., before the collapse of the local population.

### 2.3. Ethics Statement

All applicable international and national guidelines for the care and use of animals were followed. All procedures conform to the ASAB/ABS Guidelines for the Treatment of Animals in Behavioural Research and Teaching [31]. The research protocols for this study were approved by the Animal Ethics Commission of Severtsov Institute of Ecology and Evolution (protocol ## 19-2019 and 58-2022).

### 2.4. DNA Extraction and Amplification

Total genomic DNA was extracted from ethanol-preserved toe clippings (see Table S1 for sample sizes) using a standard protocol of proteinase K digestion, phenol–chloroform deproteinization, and cold ethanol precipitation [32]. The two mitochondrial loci, complete cytochrome b gene (*Cytb*, 1144 bp) and control region (*D-loop*, 934 bp), were used as genomic markers. *Cytb* was amplified using the primer pair CytB1_Mmer_F (5′-CGACGCATCCTGCTAAAAAT-3′) and CytB1_Mmer_R (5′-GGGATTTTGTCCTCCCTTCT-3′), which were designed using the full mitochondrial genome sequence from NCBI (Reference Sequence: NC_027684.1). The *D-loop* primer pair L0702mod (5′-TCCCCACCRYCRGCRCCCAAAGC-3′) and H0702 (5′-TCTAAGCATTTTCAGTGCTTTGC-3′) from [33] was modified for better amplification results. The polymerase chain reaction was carried out using 0.1–0.2 μg template DNA. The 25 µL reaction mix consisted of 15 pmol of each primer, the mixture of four dNTP (200 μM of each), 2 mM MgCl_2_, 2 units of recombinant Taq polymerase of low concentration, and 1 × PCR buffer (20 mM (NH_4_)SO_4_, 50 mM Tris-HCL (pH 8.9), 20 μM EDTA, and 150 μg/mL bovine serum albumin). Cycling conditions (T100 Thermal Cycler, BioRad, Hercules, CA, USA) involved an initial denaturation step at 95 °C for 3 min, followed by 30 cycles, which included denaturation (20 s at 95 °C), primer annealing (20 s at 58 °C for *Cytb* or 60 °C for *D-loop*), elongation (80 s for *Cytb* and 65 s for *D-loop* at 72 °C), and a final elongation (5 min at 72 °C) steps. The PCR products were visualized on a 6% polyacrylamide gel and then cut off and purified using STE (NaCl-Tris/HCl-EDTA) elution and ethanol precipitation. After purification, amplified DNA were sequenced on an ABI PRISM 3500 (Thermo Fisher Scientific, Waltham, MA, USA) genetic analyzer with a cycle sequencing kit (The BigDye^®^ Terminator v3.1, Applied Biosystems, Foster City, CA, USA,) and the same primers (used for PCR) as forward and reverse. The sequences were read and corrected using BioEdit 7.1.3.0 and aligned by the ClustalW algorithm in MEGA XI (version 11.03.13) [34] with subsequent manual fine-tuning.

### 2.5. Data Analysis

We analyzed haplotype composition and genetic diversity at three spatial scales. First, we assessed genetic diversity in each mitochondrial marker for the Eastern and Western zones combined (hereinafter referred to as the “current population”). Second, we assessed genetic diversity within and between subpopulations: “the Eastern”, “the Northeastern”, and “the Western”. At this stage, we included in the analysis samples collected in the Western zone in 2008 before the collapse of the local population (denoted as “the Western Old”). Finally, we analyzed the variation of haplotype composition across sites—the groups of neighboring patches inhabited by gerbils—within subpopulations: seven sites in the Western, five in the Eastern, three in the Northeastern, and three in the Western Old subpopulations (Table S1). We examined 138 *Cytb* sequences and 144 *D-loop* sequences for the current population and 29 *Cytb* sequences and 37 *D-loop* sequences for the Western Old subpopulation. To maintain sample sizes, we used separate markers instead of concatenated ones. Unique *Cytb* (1144 bp) and *D-loop* (934 bp) sequences of *M. meridianus* were submitted to GenBank (accession numbers: PP763760 to PP763789 for the *Cytb* data; PP763791 to PP763829 for the *D-loop* data; Table S2). Given the relatively small sample sizes, non-recombinant genetic marker, and small number of segregating sites (see Results, Table 1), we used the most appropriate [35] Fu’s F_s_ and Tajima’s D* statistics to check for an excess or a deficit of rare alleles in the analyzed samples. Calculations were performed using DnaSP software version 6.12.03 [36].

Genetic distances were calculated to quantify sequence divergences among individuals using Kimura’s two-parameter (K2P) distances model, as implemented in MEGA XI [34]. The K2P distance is the most effective model when genetic distances are supposed to be low (as at an intraspecific level) [37]. Phylogenetic trees for each locus were constructed using the maximum likelihood (ML) method implemented in MEGA XI. The best substitution models were selected based on the Bayesian information criterion (BIC), and nodal supports were assessed with non-parametric bootstrapping with 1000 replicates. The Hasegawa–Kishino–Yano model (HKY, −lnL = 1875.96, BIC = 5760.67) was selected for *Cytb* (BIC = 7760.65; −lnL = 1818.29), and the same one with a discrete Gamma distribution (HKY + G, −lnL = 1542.89, BIC = 5749.17) for *D-loop*. To assess spatial patterns of haplotype diversity, we performed Templeton–Crandall–Sing network analysis (TCS) [38,39] in PopArt v. 1.7. [40].

Haplotype compositions of subpopulations and sites within subpopulations were visualized on a map with QGIS 3.36.0. Geographic distances between sites within subpopulations were calculated using the “matrix of distances” instrument implemented in QGIS 3.36.0. We used Weir and Cockerham’s modified *F*_ST_ [41] implemented in Arlequin 3.5.2 [42] to quantify the genetic structuring between and within subpopulations. Pairwise genetic distances between sites were estimated with *F*_ST_ and Chord-normalized Euclidian distances [43,44] as implemented in PAST software (version 3.25) [45] based on haplotype frequencies. The negative *F*_ST_ values were replaced with zeros and interpreted as indicating the lack of clear genetic structure [46]. Also, the negative *F*_ST_ values may be a warning sign of sample sizes being too small relative to the number of alleles [47]. Correlations between pairwise *F*_ST_ and geographical distances (ln-transformed) were calculated for each molecular marker in R with the *mantel* function in the *vegan* package [48]. To assess genetic variation among and within current subpopulations, we performed the analysis of molecular variance (AMOVA) based on pairwise differences (using haplotype frequencies) with 1023 permutations implemented in Arlequin version 3.5.2 [42]. To compare the distributions of haplogroups between subpopulations, we used Chi-square test.

## 3. Results

### 3.1. Genetic Diversity in the Current Population

In total, we identified 29 *Cytb* haplotypes, among which 24 haplotypes were found in the current population, while five came from the Western Old subpopulation sampled in 2008. Thirty *D-loop* haplotypes were found in the current population, and nine more were private for the Western Old subpopulation. Only 21% of the *Cytb* (5 of 24) and 27% of the *D-loop* haplotypes (8 of 30) present in the current population were shared between two or more subpopulations (Table S3); the rest were subpopulation-specific.

Both markers showed high haplotype diversity, whereas the values of nucleotide diversity were less than 0.01 (Table 1), as expected for intra-population data. Haplotype and nucleotide diversity and the average number of nucleotide differences were higher at the *D-loop* region. Statistically significant negative values of Fu’s F_s_ and negative values of Tajima’s D (though low and not statistically significant at α = 0.05) in both loci indicate an excess of low-frequency haplotypes relative to what was expected under the neutral model.

**Figure 3:**
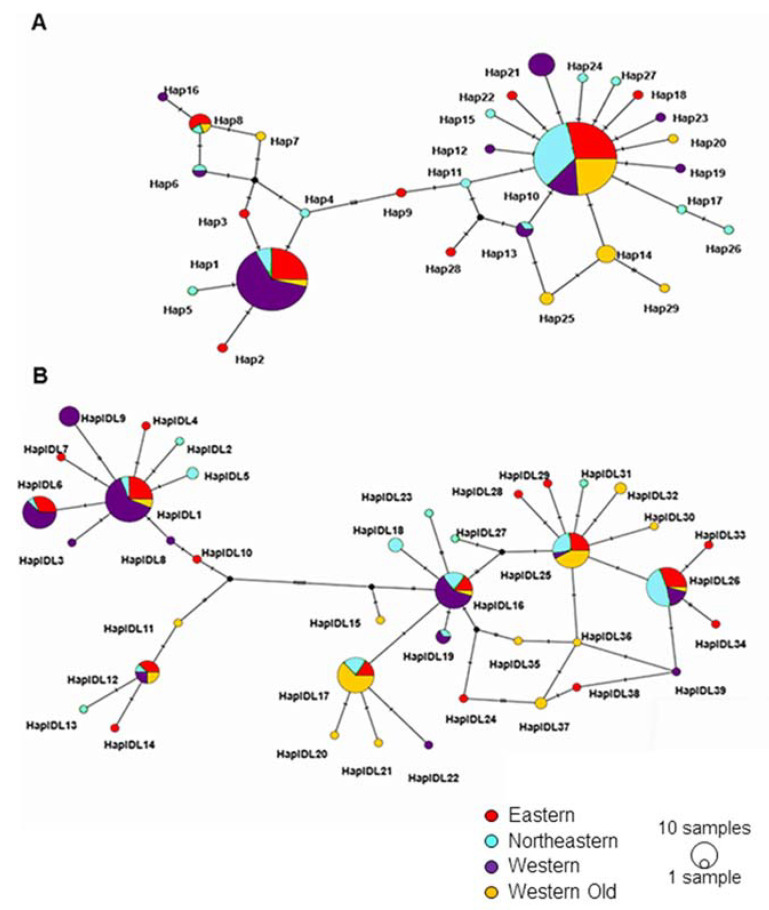
TCS haplotype network of all examined *Cytb* (**A**) and *D-loop* (**B**) sequences. Each circle represents a unique haplotype; the size of the circle is proportional to haplotype frequency. Colors represent the subpopulations. Small black circles represent hypothetical haplotypes. Hatches on the internodes show nucleotide substitutions.

Phylogenetic and network analyses identified two clades (haplogroups) for each locus (Figure S1 and Figure 3). The network analysis of *Cytb* shows that haplogroup 1 was centered around Hap1 and haplogroup 2 around Hap10 (Figure 3A). The TCS network of *D-loop* sequences had a more complex structure. Here, haplogroup 1 had two star-like structures, one centered around HaplDL1 and the other around HaplDL12. Haplogroup 2 had a network rather than a star-like shape (Figure 3B). Networks show that two haplogroups are not separated in space but differ in the frequencies of individual haplotypes.

### 3.2. Genetic Diversity and Differentiation between Subpopulations

**Figure 4:**
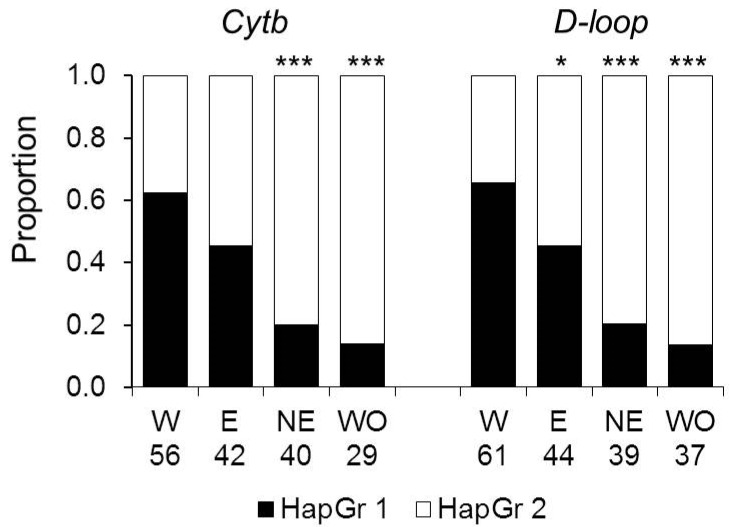
The distribution of haplogroups HapGr 1 and HapGr 2 across subpopulations. W—the Western, E—the Eastern, NE—the Northeastern, and WO—the Western Old subpopulations. The numbers show sample sizes for each subpopulation. Asterisks show significant differences from the Western subpopulation by the Chi-square test: *—*p* < 0.05, ***—*p* < 0.0001.

The proportions of haplotypes representing haplogroups 1 and 2 differed between subpopulations. For both loci, haplogroup 1 haplotypes dominated in the Western colonizing subpopulation and the most uniform distribution was in the Eastern subpopulation, and haplogroup 2 prevailed in the Northeastern and Western Old subpopulations (Figure 4). The proportion of the haplogroup 1 haplotypes in the Western colonizing population was significantly higher than in the Eastern subpopulation for *D-loop* and in the Northeastern and Western Old subpopulations for both markers.

The Eastern subpopulation had the highest haplotype and nucleotide diversity of the *Cytb* and *D-loop* sequences (Table 2). For the Northeastern subpopulation, we detected statistically significant negative values of F_s_, indicating an excess of rare alleles in both markers. The colonizing Western subpopulation had a relatively low number of haplotypes and a reduced haplotype diversity of *Cytb* compared to the Eastern and Western Old subpopulations but not the Northeastern subpopulation. The number of *D-loop* haplotypes and their diversity in the Western subpopulation were the lowest compared to all other subpopulations. The Western subpopulation had the highest positive values of F_s_ for both markers; for the *D-loop*, the deviation from the neutral model was statistically significant, indicating a deficit of rare alleles. The Western Old subpopulation exhibited an excess of rare alleles in both markers (significant for the *D-loop* region) and the lowest values of nucleotide diversity combined with relatively high haplotype diversity. Thus, despite relatively high haplotype diversity, the haplotypes within this sample were similar (Figure 2).

**Figure 5:**
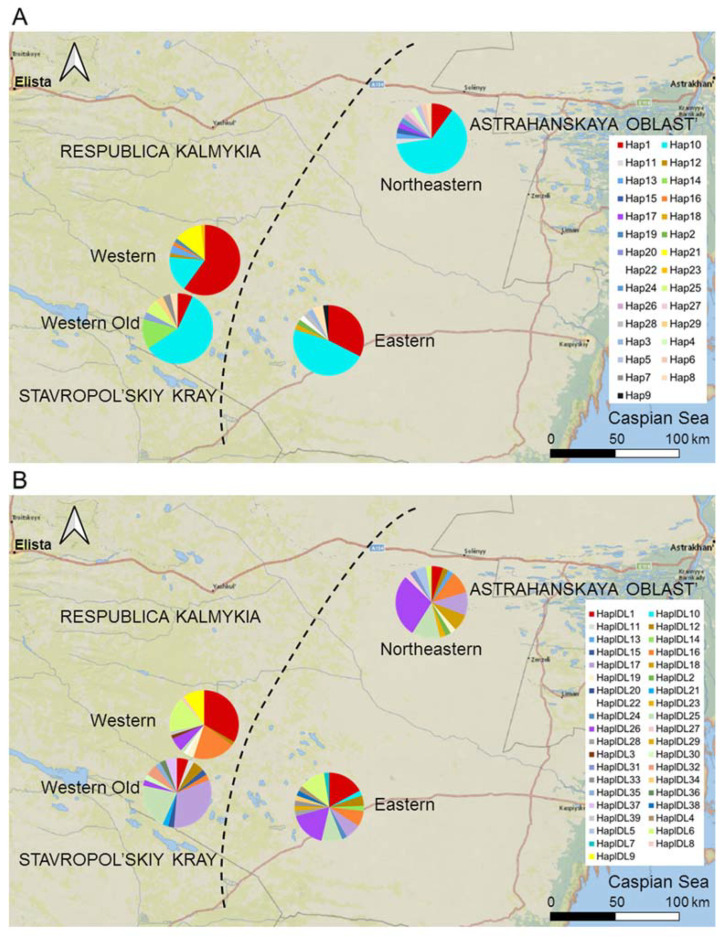
The distribution of haplotypes of (**A**) *Cytb* and (**B**) *D-loop* in the subpopulations of the midday gerbil. The Western subpopulation is a colonizing population, the Eastern and Northeastern subpopulations are core subpopulations, and the Western Old subpopulation represents the Western population of gerbils before its collapse in 2017. The diagram for the Western Old population is shifted to the southwest to not overlap with the current Western population. See Table S1 for sample sizes.

Although the proportion of private haplotypes in the colonizing Western subpopulation was relatively low, its haplotype frequencies differed from those in the Eastern, Northeastern, and Western Old subpopulations (Figure 5). In particular, the proportions of the *Cytb* Hap1 haplotype; the *D-loop* haplotypes HapDL1 and HapDL6; and, rare in other subpopulations, HapDL16, were higher, whereas the proportion of HapDL26 was lower than in the core subpopulations.

AMOVA revealed significant differentiation among and within current subpopulations for both markers (*p* < 0.0001), with the larger proportion of molecular genetic variation attributed to differences within subpopulations (85.9% and 85.2% for *Cytb* and *D-loop*, respectively). The overall *F*_ST_ was 0.14 and 0.15 for *Cytb* and *D-loop*, respectively. Kimura2-parameter genetic distances between subpopulations in both loci were less than 1%, corresponding to the within-population level of variation (Table 3). Pairwise *F*_ST_ values varied considerably, ranging from 0.008 to 0.331 for *Cytb* and from 0.012 to 0.337 for *D-loop*. Strong differentiation was observed between the subpopulations that differed greatly in the proportions of *Cytb* and(or) *D-loop* haplogroups (Figure 4, Table 3). Thus, within the current population, the *F*_ST_ value was large and was the largest (for both *Cytb* and *D-loop* loci) between the Western and the Northeastern subpopulations and the smallest between the Western and the Eastern subpopulations, in agreement with the differences in the proportions of haplogroups. Other metrics of genetic differences also indicated that the Western subpopulation of colonists is genetically much more similar to the Eastern than to the Northeastern subpopulation. This pattern was expressed more clearly for *D-loop* haplotypes, whereas for *Cytb* haplotypes, the Western subpopulation was only slightly closer to the Eastern than to the Northeastern subpopulation. The extinct Western Old subpopulation was most similar to the current Northeastern subpopulation and most distant from the current colonizing Western subpopulation (Table 3).

### 3.3. Genetic Diversity and Differentiation within the Colonizing Subpopulation

**Figure 6:**
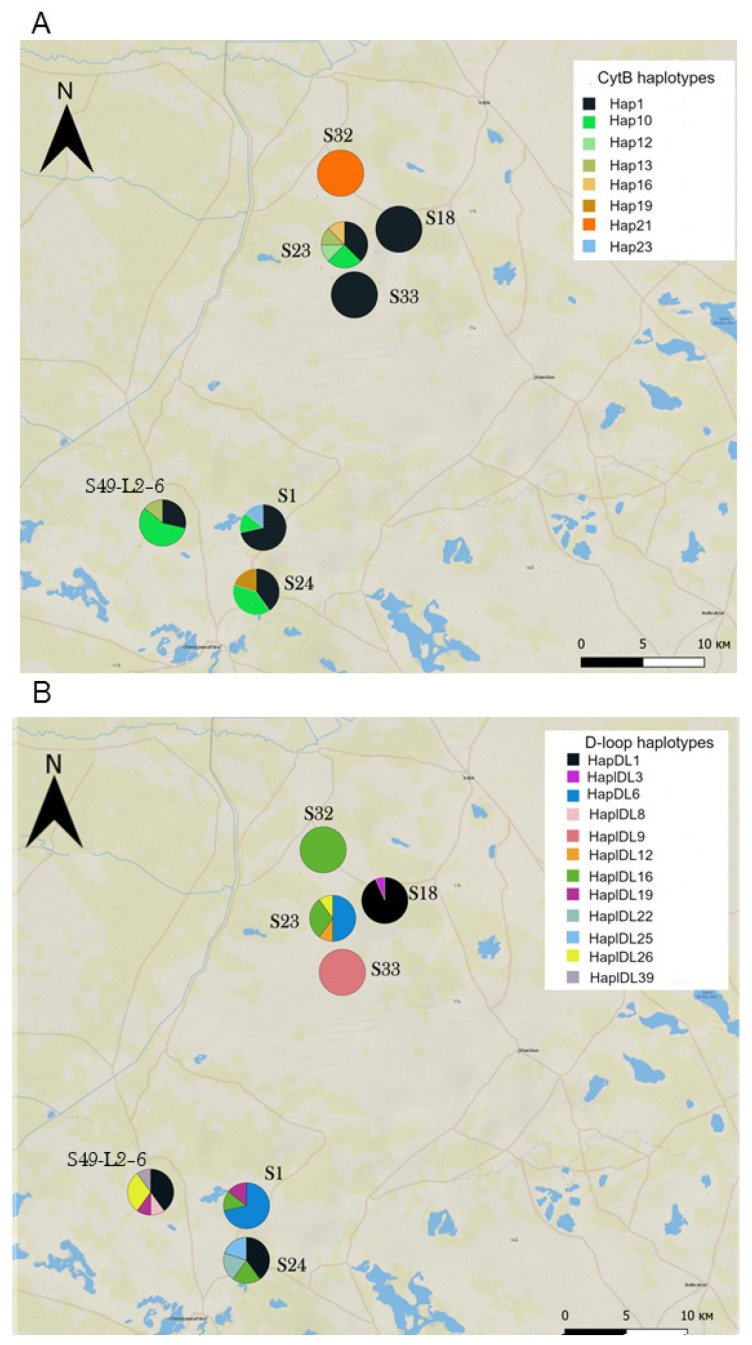
Haplotype composition of sites within the colonizing Western subpopulation. (**A**) *Cytb* haplotypes, (**B**) *D-loop* haplotypes. See Table S1 for sample sizes.

Haplotype composition and diversity varied considerably between sites within the colonizing population—in some of them, gerbils were monomorphic at one (S18, the site sampled most intensively, Table S1) or both loci (S32 and S33); in the others, they showed a high diversity of haplotypes (Figure 6). In some “monomorphic sites”, gerbils carried widespread haplotypes (e.g., *Cytb* Hap1 in S33 and S18 and *D-loop* HapDl16 in S32), while in others they were monomorphic for haplotypes exclusive for the Western subpopulation (*Cytb* Hap21 in S32 and *D-loop* HapDL9 in S33). Furthermore, gerbils from neighboring sites could be monomorphic for the same or different haplotypes. The Mantel test revealed no correlation between pairwise geographical distance and *F*_ST_ either for *Cytb* or for *D-loop* (*r* = 0.09, *p* = 0.3 and *r* = 0.01, *p* = 0.4, respectively). Sites within the Western subpopulation showed a high average level of differentiation as well as substantial variation in pairwise genetic distances in contrast to sites within the core population (Eastern and Northeastern subpopulations combined), as shown by the larger chord-normalized Euclidian distances, the much higher and mostly significant *F*_ST_ values, and their larger variances (Table 4 and Table S4). The Western Old subpopulation occupied the intermediate position, being less spatially structured than the current Western subpopulation and more differentiated than the core population.

## 4. Discussion

### 4.1. The Midday Gerbil Population Shows Signs of Demographic and Spatial Expansion

In our study, we attempted to make a “snapshot” of haplotype diversity in a population of the midday gerbil, which is now re-colonizing previously occupied areas. This allowed us to study the changes in the genetic composition of the colonizing population compared to the core populations at the first steps of colonization.

The whole studied population exhibits relatively high levels of haplotype diversity. High values of haplotype diversity and the low proportion of haplotypes shared between subpopulations indicate a high level of genetic differentiation. Combined with the excess of rare alleles, this is consistent with a theoretical model of expanding population [9].

Along with the re-emergence of the desert habitats due to recent desertification [30], the current re-colonization of the westernmost areas is likely a result of not only spatial but also demographic expansion of the Eastern core population, indicated by the statistically significant negative Fu’s Fs values [49]. The nucleotide diversity for both markers was low, indicating the similarity of haplotypes within the population. Nevertheless, the network analysis detected two well-solved clusters of haplotypes in both markers, which also fits well with the model of spatially and demographically expanding populations [9,35].

Based on genetic distances and the level of differentiation between current subpopulations (Table 3), the colonizing subpopulation is much more similar to the Eastern than to the Northeastern core subpopulation, suggesting that the former is the main source of colonization. The genetic distance and differentiation are the greatest between the current and the extinct Western subpopulations and minimal between the extinct and the current Northeastern subpopulation. Thus, the current Western subpopulation is much more likely a result of recolonization from the core Eastern population rather than a recovery of the residual population that survived in situ after the population collapse.

### 4.2. Genetic Specificity of the Recently Established Colonist Subpopulation

The Western colonizing subpopulation differed substantially from the core Eastern subpopulations and the extinct Western Old subpopulation in the proportions of haplogroups 1 and 2 (Figure 4). In the colonizing subpopulation, the distribution was strongly biased towards haplogroup 1 for both markers, whereas in eastern subpopulations and the extinct Western Old subpopulation, it was biased towards haplogroup 2. The biased haplogroup ratio contributing to differentiation between the colonizing and the source population suggests spatial sorting of haplotypes at the expansion edge, consistent with the theoretical predictions and some empirical studies showing spatial sorting of genotypes and phenotypes on the wave of expansion [12,50,51,52,53].

We did not detect substantial decrease in the *cytochrome b* haplotype diversity in the colonizing subpopulation compared to the core population: It was lower than in the Eastern but higher than in the Northeastern subpopulation. However, *D-loop* haplotype diversity in colonists was lower than in both core subpopulations and the extinct Western Old subpopulation (Table 2). In addition, Fu’s Fs was positive for both markers and, for the *D-loop*, it was statistically significant at *p* < 0.1 (here, *p* = 0.06), indicating a loss of rare alleles. This pattern is typical of the colonizing populations under the model of the founder effect [2]. Therefore, our results support the founder effect hypothesis predicting reduced genetic diversity in the colonizing population of gerbils.

On the other hand, the haplotype diversity in the colonizing population, though lower than in the core population, was still high, especially for *D-loop*. Indeed, the probability that two random colonists carry different haplotypes was about 0.6 for cytochrome b and 0.8 for *D-loop* (Table 2). The high haplotype diversity in the colonizing population can be explained by the ongoing gene flow from the source population, the genetic admixture from multiple sources, or both, which can compensate for the loss in genetic diversity caused by the founder effect [2,14,15,16].

We also observed changes in the haplotype composition in the colonists compared to the core population. In particular, we (i) revealed differences in the frequencies of common and rare alleles and (ii) identified haplotypes specific to the colonizing population (Figure 5, Table 2). The first pattern was especially pronounced for the *D-loop* haplotypes HaplDL1 and HaplDL16, which comprised the majority of samples in the colonizing population but were rare elsewhere (Figure 5B). The increased proportions of relatively rare haplotypes among colonists are consistent with the hypothesized allele-surfing effect on the genetic diversity of the colonizing populations [2,8,9,54,55].

So far, allele surfing, as a specific characteristic of expanding populations, has been predicted from theoretical models and was mainly observed in the microcosm or experiments [7,8,9]. Few studies have revealed the allele-surfing signatures in wild populations [2]. In the common vole expanding its range in Ireland, the genetic diversity decreased along the expansion axis [11]. In invasive tortoises in Spain, allele surfing was signified by reduced genetic diversity and clinal patterns in allele distributions combined with higher spatial differentiation [10]. The midday gerbil in Kalmykia provides an additional example of allele surfing in an expanding population.

Allele surfing may be manifested in particular in a relatively high proportion of private haplotypes in the colonizing population of gerbils. The majority of these are singletons, thus found in a single individual; however, the *Cytb* Hap21 haplotype was found in 12.5% of colonists (7 of 56) and the *D-loop* HaplDL9 in 8% (5 of 61). These alleles could be rare and, thus, not sampled in the core population. Alternatively, the absence of these haplotypes in the core population may suggest an unknown source of expansion, possibly resulting from long-distance dispersal from distant sources [2,56], thus, explaining the high frequencies of private haplotypes in the colonizing population. Therefore, we cannot reject either their surfing from the core population on the wave of expansion or their arrival from the unknown source of expansion.

More solid evidence for allele surfing comes from a stronger genetic structure of the colonizing population compared to the core population, as indicated by the considerably lower genetic similarity between sites and large and mostly significant pairwise *F*_ST_ values despite much shorter inter-site geographical distances (Table 4). Together with a strong differentiation between the colonizing and core population, this pattern is consistent with the allele-surfing theory, which predicts the genetic structuring of not only newly colonized areas but also the whole [2,8,9] population.

One more specific feature of the recently established colonizing population is that in some sites, gerbils were genetically monomorphic at one or both loci (Figure 6). Moreover, gerbils from neighboring sites could be monomorphic for different haplotypes, indicating spatial segregation at a fine scale. The lack of correlation between geographical and genetic distances confirms that the sites spatially close to each other could differ substantially in haplotype composition and frequency. Together with high spatial differentiation, this implies low gene flow and low genetic admixture between newly founded demes on the wave of the midday gerbil’s range expansion.

Reduced genetic diversity and high genetic differentiation caused by allele surfing combined with low gene flow between demes of colonists is a characteristic of the early stages of colonization in the expanding ranges. This is a transient state, and with time post-colonization, the increasing interdeme migration along with the influx of migrants from the core populations are expected to weaken genetic structure and increase genetic diversity [2,8,9,14]. The colonizing population of the midday gerbil is now rapidly increasing and expanding, and the ongoing development and expansion of the burrow network increases the landscape connectivity [28,30], which should neutralize the founder and allele-surfing effects and lower the differences in genetic structure between the core and the edge by increasing genetic diversity and weakening spatial differentiation. On the other hand, the transient strong genetic structure at the leading edge may last for long periods in species with low dispersal abilities [9]. Female midday gerbils are strongly philopatric and site-attached [57], which may limit gene flow, maintaining strong differentiation of the colonizing population for a long period.

Higher fragmentation and lower connectivity of habitats at range margins may also contribute to maintaining strong population differentiation of the Western population, as suggested by the theory of species distribution modelling [58,59]. Genetic differentiation can be caused by the isolation of demes and subpopulations at range margins due to the low rates of interpatch movements, as demonstrated in numerous studies (e.g., [60,61]. In support of these theoretical and empirical studies, we found a pronounced genetic differentiation between sites in the extinct marginal Western subpopulation, which has persisted here from at least the 1980s until its collapse in 2017. Moreover, in terms of genetic diversity and spatial structuration, the Western Old subpopulation occupies the intermediate position between the current Western colonizing subpopulation and the core population, suggesting that reduced genetic diversity and increased genetic differentiation are general features of marginal populations, enhanced by the founder and allele-surfing effects at the leading edges of expanding ranges.

## 5. Conclusions

Human-induced landscape transformations cause range shifts and expansions, which can have significant ecological and evolutionary consequences. Our study of the desert rodent currently expanding its range in response to human-induced desertification of rangelands in southern Russia revealed genetic differences between the colonizing and the core population. In the colonizing population, we found reduced genetic diversity, the redistribution of haplotype frequencies—in particular, in favor of variants rare in the core population—and strong genetic structure combined with strong differentiation from the core population—genetic patterns consistent with allele-surfing theory supported so far in a few studies of terrestrial vertebrates. In terms of genetic diversity and spatial structuration, the edge population of gerbils before its collapse occupies the intermediate position between the current colonizing and core populations. We conclude that reduced genetic diversity and increased genetic differentiation are general features of marginal populations, enhanced by the founder and allele-surfing effects at the leading edges of expanding ranges. Our findings provide new insights into understanding genetic processes, which accompany range expansions, as well as the genetic consequences of colonization.

## Figures and Tables

**Table 1:** Genetic diversity in the current population of the midday gerbils, *Meriones meridianus,* based on *Cytb* and *D-loop* sequences.

Locus	*N*	h	S	Eta	Hd	Pi	k	V	Fu’s F_s_	Tajima’s D
*Cytb*	138	24	23	23	0.716	0.0031	3.554	0.0007	−6.45 *	−0.426 *p* > 0.10
*D-loop*	144	30	32	32	0.896	0.0080	7.468	0.0001	−2.92 *	−0.164 *p* > 0.10

*N—*number of sequences; h—number of haplotypes; S—number of polymorphic (segregating) sites; Eta—total number of mutations; Hd—haplotype diversity; Pi—nucleotide diversity; k—average number of nucleotide differences; V—variance of haplotype diversity. *—Statistically significant at *p* < 0.05.

**Table 2:** Genetic diversity within four subpopulations of *Meriones meridianus*.

Subpopulation	*N*	h	S	Hd	Pi	hpr	*N*pr/*N*	Fu’s F_s_	Tajima’s D
	*Cytb*								
Eastern	42	9	12	0.685	0.003	6	0.14	0.703	0.879
Northeastern	40	13	15	0.608	0.002	8	0.20	−4.077 ***	−0.905
Western	56	9	14	0.619	0.003	5	0.20	1.078	0.349
Western Old	29	8	13	0.645	0.002	5	0.31	−0.739	−0.915
	*D-loop*								
Eastern	44	17	23	0.918	0.008	10	0.23	−0.903	1.420
Northeastern	39	15	25	0.889	0.007	7	0.26	−1.132 *	0.139
Western	61	12	23	0.823	0.007	5	0.16	2.383 **	0.983
Western Old	37	15	22	0.846	0.005	9	0.30	−2.260 ***	−0.148

*N*—the number of sequences; h—the number of haplotypes; S—the number of polymorphic (segregating) sites; Hd—haplotype diversity; Pi—nucleotide diversity; hpr—the number of private (found only in the given subpopulation) haplotypes; *N*pr—the number of sequences of private haplotypes; *—*p* = 0.1, **—*p* < 0.1, ***—*p* < 0.05.

**Table 3:** Genetic distances between subpopulations of *Meriones meridianus*.

Subpopulation 1	Subpopulation 2	K_1_	K_2_	K_1,2_	*F*_ST_	Dxy	Da	K2P
*Cytb* Current subpopulations								
Northeastern	Western	2.524	3.405	4.034	0.265	0.0035	0.00094	0.0035
Eastern	Western	3.592	3.405	3.688	0.051	0.0032	0.00017	0.0032
Northeastern	Eastern	2.524	3.592	3.321	0.079	0.0029	0.00023	0.0029
*Cytb* Current subpopulations versus extinct subpopulation								
Eastern	Western Old	3.592	2.399	3.489	0.141	0.0031	0.00043	0.0032
Northeastern	Western Old	2.524	2.399	2.482	0.008	0.0022	0.00002	0.0022
Western	Western Old	3.405	2.399	4.336	0.331	0.0038	0.00125	0.0038
*D-loop* Current subpopulations								
Northeastern	Western	6.157	6.449	8.616	0.268	0.0092	0.00247	0.0025
Eastern	Western	7.570	6.449	7.516	0.067	0.0080	0.00054	0.0005
Northeastern	Eastern	6.157	7.570	7.400	0.073	0.0079	0.00057	0.0006
*D-loop* Current subpopulations versus extinct subpopulation								
Eastern	Western Old	7.570	5.081	7.345	0.139	0.0078	0.00109	0.0011
Northeastern	Western Old	6.157	5.081	5.686	0.012	0.0061	0.00007	0.0001
Western	Western Old	6.449	5.081	8.701	0.337	0.0093	0.00313	0.0032

K—the average number of nucleotide differences within (K_1_ and K_2_) and between (K_1,2_) subpopulations; *F*_ST_—pairwise fixation index; Dxy—nucleotide divergence: the average number of nucleotide substitutions per site between subpopulations; Da—net genetic distance: the number of net nucleotide substitutions per site between subpopulations; K2P—Kimura-2-Parameter genetic distance.

**Table 4:** Spatial differentiation within the colonizing (Western), Western Old, and core subpopulations (Eastern and Northeastern subpopulations combined). Average pairwise geographical distances, *F*_ST_ values, and chord-normalized genetic Euclidian distances.

Population	Distance, km (min–max)	*F*_ST_ (min–max)	Variance *F*_ST_	P	Chord (min–max)	Variance Chord
	*Cytb*					
Western	18.6 (1.1–33.7)	0.389 (0.000–1.000)	0.11	0.48	0.86 (0.00–1.41)	0.19
Eastern	73.0 (12.9–148.5)	0.046 (0.000–0.359)	0.01	0.07	0.60 (0.30–1.06)	0.04
Western Old	2.7 (0.9–3.6)	0.113 (0.000–0.232)	0.01	0.67	0.91 (0.21–1.26)	0.36
	*D-loop*					
Western		0.425 (0.000–1.000)	0.11	0.81	1.22 (0.45–1.41)	0.09
Eastern		0.038 (0.000–0.282)	0.01	0.14	1.06 (0.67–1.41)	0.04
Western Old		0.216 (0.099–0.398)	0.03	0.67	1.13 (0.92–1.41)	0.06

P—the proportions of statistically significant (at *p* < 0.05) positive pairwise *F*_ST_ values.

## Data Availability

The original dataset generated and analyzed during the current study is included in the supplementary material as Supplementary Material S2 (Haplotype_data_set.xlsx).

## Supplementary Materials

The following supporting information can be downloaded at: https://www.mdpi.com/article/10.3390/ani14182720/s1, Supplementary Material S1. A collection of supplementary figures and tables (SM_1.pdf). Figure S1. Maximum-likelihood trees for the Cytb (A) and D-loop (B) datasets. Numbers near branch nodes indicate bootstrap values based on 1000 replicates (%). Table S1. Site codes, coordinates, sample sizes, and the number of identified haplotypes of *Cytb* and *D-loop* across sites and subpopulations. Table S2. GenBank accession numbers for *Cytb* and *D-loop* haplotypes. Table S3. The distribution of haptolypes of *Cytb* and *D-loop* across four subpopulations. Table S4. Pairwise geographical distances, *F*_ST_ values, and chord-normalized genetic Euclidian distances between sites within the colonizing (Western), Western Old, and the core subpopulations (Eastern and Northeastern subpopulations combined). Supplementary Material S2. Haplotype dataset (Haplotype_data_set.xlsx).
