# Supplementary material for: Does the Colonizing Population Exhibit a Reduced Genetic Diversity and Allele Surfing? A Case Study of the Midday Gerbil (Meriones meridianus Pallas) Expanding Its Range
Source: Animals (Basel). 2024 Sep 20;14(18):2720. doi: 10.3390/ani14182720 (PMC11429244; doi:10.3390/ani14182720)
Supplement: Supplementary file 1 [file animals-14-02720-s001.zip › SM_1.pdf]

Supplementary Material S1

Figures and tables

Olga N. Batova, Nikolai I. Markov, Sergei V. Titov, Andrey V. Tchabovsky

Does the colonizing population exhibit a reduced genetic diversity and allele surfing? A case study of the midday gerbil (*Meriones meridianus* Pallas) expanding its range

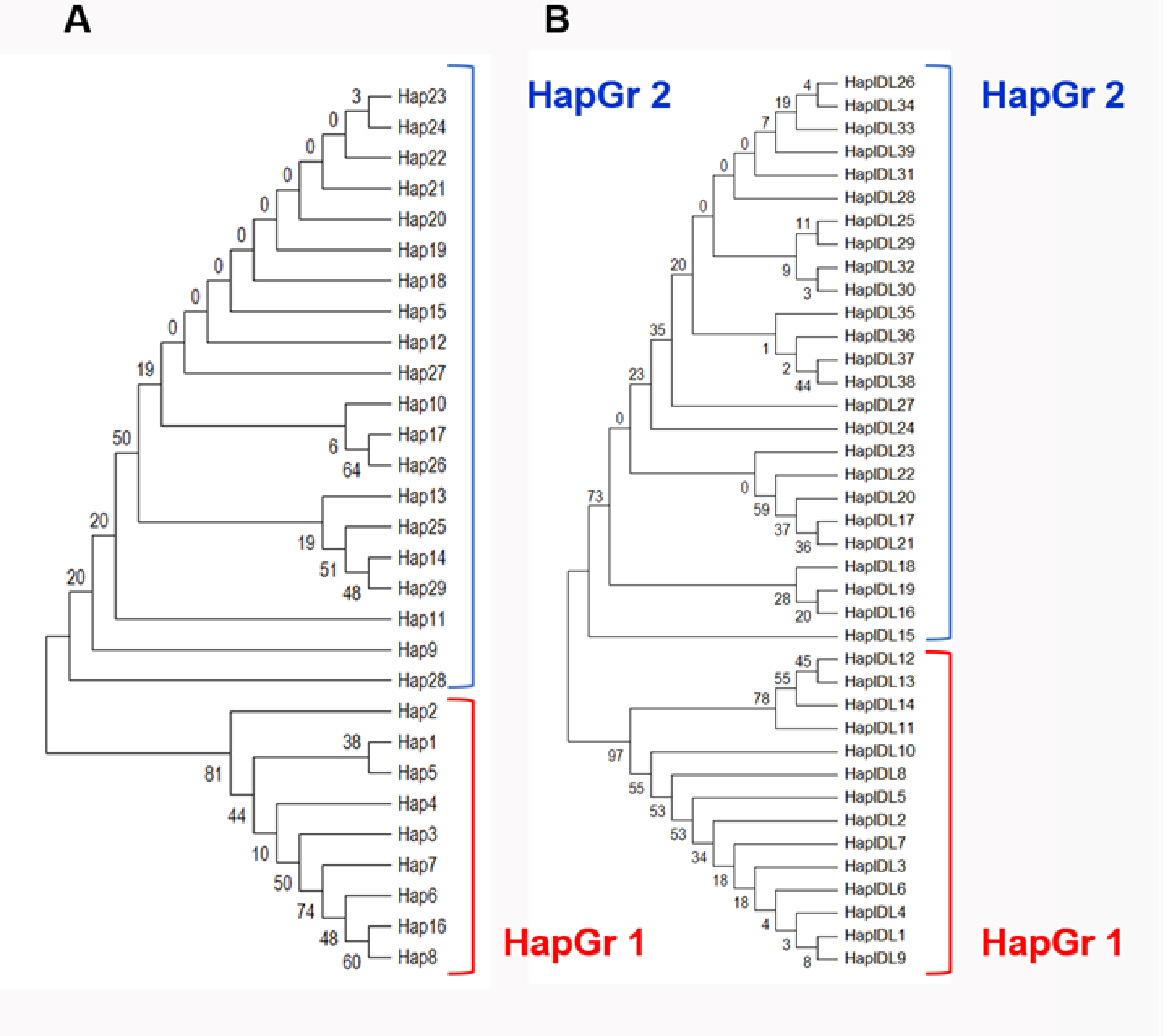

Figure S1. Maximum-likelihood trees for the *Cytb* (A) and *D-loop* (B) data sets. Numbers near branch nodes indicate bootstrap values based on 1,000 replicates (%).

Table S1. Site codes, coordinates, sample sizes and the number of identified haplotypes of *Cytb* and *D-loop* across sites and subpopulations

Site Code	Subpopulation	Lat	Long	*N* seq *Cytb*	*N Cytb* haplotypes	*N* seq *D-loop*	*N D-loop* haplotypes
			Western zone				
S49-L2-6	Western	45.4768	45.2795	7	3	10	5
S1	Western	45.4688	45.3846	7	3	7	3
S24	Western	45.4342	45.3782	5	3	5	4
S32	Western	45.7314	45.4630	7	1	7	1
S23	Western	45.6795	45.4671	8	5	10	4
S33	Western	45.6726	45.4773	6	1	6	1
S18	Western	45.6908	45.5234	15	1	15	2
S48*	Western	45.4514	45.3155	1	1	1	1
Western total				56	9	61	12
Tam	Western Old	45.4755	45.2890	10	6	16	6
Zb	Western Old	45.4794	45.2811	11	5	12	8
Tri	Western Old	45.4506	45.2599	8	1	9	4
Western Old total				29	9	37	15
			Eastern zone				
Prikumskii	Eastern	45.2490	45.8315	4	3	4	4
S46	Eastern	45.1512	45.6308	4	4	4	4
Tsuva-1	Eastern	45.4766	46.3122	7	2	8	6
S19-Koms	Eastern	45.3749	45.9979	12	4	12	8
Naryn-Khuduk	Eastern	45.4281	46.4565	13	5	13	8
Dag*	Eastern	45.0159	46.4843	1	1	1	1
Lagan'*	Eastern	45.6058	46.1042	1	2	2	2
Eastern total				42	10	44	17
S6	Northeastern	46.3082	46.3883	13	6	13	6
S44-45	Northeastern	46.2930	46.6754	17	6	17	10
S8-9	Northeastern	45.9595	46.5464	10	4	10	4
Northeastern total				40	13	39	15
	TOTAL			167	29	181	39

* excluded from the analysis of intra-subpopulation variation

N seq – the number of sequences examined

Table S2. GenBank accession numbers for *Cytb* and *D-loop* haplotypes.

Cytb		*D-loop*	
Haplotype labels	Accession #	Haplotype labels	Accession #
Hap1	PP763760	HapDL1	PP763797
Hap2	PP763771	HapDL2	PP763817
Hap3	PP763782	HapDL3	PP763799
Hap4	PP763784	HapDL4	PP763820
Hap5	PP763785	HapDL5	PP763798
Hap6	PP763786	HapDL6	PP763812
Hap7	PP763787	HapDL7	PP763824
Hap8	PP763788	HapDL8	PP763829
Hap9	PP763789	HapDL9	PP763813
Hap10	PP763761	HapDL10	PP763823
Hap11	PP763762	HapDL11	PP763801
Hap12	PP763763	HapDL12	PP763802
Hap13	PP763764	HapDL13	PP763818
Hap14	PP763765	HapDL14	PP763821
Hap15	PP763766	HapDL15	PP763811
Hap16	PP763767	HapDL16	PP763807
Hap17	PP763768	HapDL17	PP763791
Hap18	PP763769	HapDL18	PP763792
Hap19	PP763770	HapDL19	PP763795
Hap20	PP763772	HapDL20	PP763803
Hap21	PP763773	HapDL21	PP763806
Hap22	PP763774	HapDL22	PP763816
Hap23	PP763775	HapDL23	PP763819
Hap24	PP763776	HapDL24	PP763827
Hap25	PP763777	HapDL25	PP763800
Hap26	PP763778	HapDL26	PP763796
Hap27	PP763779	HapDL27	PP763793
Hap28	PP763780	HapDL28	PP763822
Hap29	PP763781	HapDL29	PP763826
		HapDL30	PP763810
		HapDL31	PP763794
		HapDL32	PP763805
		HapDL33	PP763814
		HapDL34	PP763815
		HapDL35	PP763808
		HapDL36	PP763809
		HapDL37	PP763804
		HapDL38	PP763825
		HapDL39	PP763828

Table S3. The distribution of haptolypes of *Cytb* and *D-loop* across four subpopulations shown as the number of individuals with a particular haplotype. Alleles shared between two or more subpopulations are marked with bold font.

	Cytb					*D-loop*			
	Subpopulations								
Haplotypes	E	NE	W	WO		E	NE	W	WO
**Hap1**	13	4	33	2	**HapDL1**	8	2	20	2
Hap2	1				HapDL2		1		
Hap3	1				HapDL3			1	
Hap4		1			HapDL4	1			
Hap5		1			HapDL5		2		
**Hap6**		1	1		**HapDL6**	5	1	10	
Hap7				1	HapDL7	1			
**Hap8**	3	1		1	HapDL8			1	
Hap9	1				HapDL9			6	
**Hap10**	20	25	9	17	HapDL10	1			
Hap11		1			HapDL11				1
Hap12			1		**HapDL12**	3	1	2	2
**Hap13**		1	2		HapDL13		1		
Hap14				4	HapDL14	1			
Hap15		1			HapDL15				1
Hap16			1		**HapDL16**	3	4	12	1
Hap17		1			**HapDL17**	3	4		12
Hap18	1				HapDL18		3		
Hap19			1		**HapDL19**		1	2	
Hap20				1	HapDL20				1
Hap21			7		HapDL21				1
Hap22	1				HapDL22			1	
Hap23			1		HapDL23		1		
Hap24		1			HapDL24	1			
Hap25				2	**HapDL25**	5	5	1	8
Hap26		1			**HapDL26**	7	11	4	1
Hap27		1			HapDL27		1		
Hap28	1				HapDL28	1			
Hap29				1	HapDL29	1			
					HapDL30				1
					HapDL31		1		
					HapDL32				2
					HapDL33	1			
					HapDL34	1			
					HapDL35				1
					HapDL36				1
					HapDL37				2
					HapDL38	1			
					HapDL39			1	
Total	42	40	56	29		44	39	61	37

Table S4. Pairwise geographical distances, F_{ST} values, and Chord-normalized genetic Euclidian distances between sites within the colonist (Western), Western Old, and the core subpopulations (Eastern and Northeastern subpopulations combined).

Subpopulation			*Cytb*			*D-loop*		
Site 1	Site 2	Distance, km	F_{ST}	*p*	Chord	F_{ST}	*p*	Chord
Western								
S49-L2-6	S1	8.4	0.161	0.163	0.91	0.085	**0.001**	1.39
S49-L2-6	S24	9.2	0.000	0.849	0.50	0.000	0.220	0.93
S49-L2-6	S32	31.7	0.483	**0.001**	1.41	0.418	**0.000**	1.41
S49-L2-6	S23	26.8	0.000	0.819	0.60	0.000	**0.002**	1.35
S49-L2-6	S33	26.7	0.620	**0.039**	1.06	0.473	**0.001**	1.41
S49-L2-6	S18	30.4	0.759	**0.001**	1.06	0.482	**0.001**	0.70
S1	S24	3.9	0.016	0.466	0.68	0.069	**0.022**	1.36
S1	S32	29.8	0.711	**0.000**	1.41	0.656	**0.004**	1.27
S1	S23	24.3	0.000	0.680	0.60	0.000	0.730	0.45
S1	S33	23.8	0.125	1.000	0.27	0.472	**0.002**	1.41
S1	S18	26.9	0.304	0.091	0.27	0.408	**0.000**	1.41
S24	S32	33.7	0.519	**0.001**	1.41	0.322	**0.010**	1.12
S24	S23	28.1	0.000	1.000	0.58	0.000	**0.023**	1.27
S24	S33	27.6	0.513	0.063	0.82	0.590	**0.002**	1.41
S24	S18	30.7	0.701	**0.008**	0.82	0.669	**0.009**	0.70
S32	S23	5.8	0.471	**0.001**	1.41	0.450	**0.012**	1.00
S32	S33	6.6	1.000	**0.001**	1.41	1.000	**0.001**	1.41
S32	S18	6.5	1.000	**0.000**	1.41	1.000	**0.000**	1.41
S23	S33	1.1	0.302	0.197	0.71	0.426	**0.001**	1.41
S23	S18	4.6	0.478	**0.001**	0.71	0.400	**0.000**	1.41
S33	S18	4.1	0.000	1.000	0.00	1.000	**0.000**	1.41
Average		*18.6*	*0.389*	*10*[a]	*0.861*	*0.425*	*17*[a]	*1.22*
Min		*1.1*	*0.0*	*21*[b]	*0.0*	*0.000*	*21*[b]	*0.45*
Max		*33.7*	*1.0*	*0.48*[c]	*1.4*	*1.000*	*0.81*[c]	*1.41*
Variance		*141.1*	*0.11*		*0.19*	*0.11*		*0.09*

Eastern+Northestern

Site 1	Site 2	Distance, km	F_{ST}	*p*	Chord	F_{ST}	*p*	Chord
Prikumskii	S46	17.2	0.000	1.000	0.816	0.000	1.000	1.000
Prikumskii	Tsuva-1	47.0	0.000	0.480	0.451	0.000	0.654	1.095
Prikumskii	S19-Koms	19.6	0.000	0.701	0.456	0.000	0.623	1.056
Prikumskii	Naryn-Khuduk	54.4	0.088	0.506	0.677	0.014	0.570	1.121
Prikumskii	S6	125.9	0.000	0.841	0.500	0.000	0.129	1.274
Prikumskii	S44-45	133.9	0.000	0.403	0.572	0.000	0.745	1.019
Prikumskii	S8-9	97.3	0.000	0.462	0.644	0.007	0.165	0.944
S46	Tsuva-1	64.1	0.000	0.126	0.833	0.000	0.274	1.414
S46	S19-Koms	36.4	0.000	0.318	0.724	0.000	0.822	1.188
S46	Naryn-Khuduk	71.6	0.147	0.185	1.058	0.041	0.683	1.035
S46	S6	139.0	0.000	0.515	0.775	0.000	0.315	1.115
S46	S44-45	148.5	0.000	0.422	0.628	0.000	0.980	0.748
S46	S8-9	112.8	0.000	0.464	0.644	0.022	**0.037**	1.364

Tsuva1	S19-Koms	27.9	0.000	0.860	0.299	0.000	0.542	0.762
Tsuva1	Naryn-Khuduk	12.9	0.000	0.906	0.408	0.034	0.195	1.142
Tsuva1	S6	91.9	0.000	1.000	0.338	0.000	0.081	1.223
Tsuva1	S44-45	94.2	0.154	0.372	0.573	0.114	0.135	1.109
Tsuva1	S8-9	55.9	0.031	0.094	0.668	0.000	0.208	0.666
S19-Koms	Naryn-Khuduk	37.0	0.055	0.459	0.603	0.006	0.543	0.922
S19-Koms	S6	108.5	0.000	1.000	0.345	0.000	0.149	1.104
S19-Koms	S44-45	115.2	0.074	0.365	0.400	0.083	0.753	0.823
S19-Koms	S8-9	78.1	0.000	0.322	0.458	0.027	**0.020**	0.754
Naryn-Khuduk	S6	97.9	0.093	0.410	0.618	0.045	0.307	0.936
Naryn-Khuduk	S44-45	97.4	0.359	**0.013**	**0.898**	0.282	0.097	1.160
Naryn-Khuduk	S8-9	59.3	0.227	**0.021**	**0.957**	0.228	**0.006**	1.260
S6	S44-45	22.2	0.056	0.204	0.494	0.041	**0.057**	1.155
S6	S8-9	40.6	0.000	0.252	0.580	0.066	**0.002**	1.257
S4445	S8-9	38.4	0.000	0.660	0.302	0.052	0.092	0.989
Average		*73.0*	*0.046*	*2*[a]	*0.60*	*0.038*	*4*[a]	*1.06*
Min		*12.9*	*0.000*	*28*[b]	*0.30*	*0.000*	*28*[b]	*0.67*
Max		*148.5*	*0.359*	*0.07*[c]	*1.06*	*0.282*	*0.14*[c]	*1.41*
Variance		*1647.6*	*0.007*		*0.04*	*0.005*		*0.04*

Western Old

Site 1	Site 2	Distance, km	F_{ST}	*p*	Chord	F_{ST}	*p*	Chord
Tam	Zb	0.9	0.106	**0.002**	1.245	0.099	**0.004**	1.067
Tam	Tri	3.6	0.232	**0.001**	1.262	0.398	**0.000**	1.414
Zb	Tri	3.6	0.000	1.000	0.213	0.151	0.177	0.923
Average		*2.7*	*0.113*	*2*[a]	*0.91*	*0.216*	*2*[a]	*1.13*
Min		*0.9*	*0.000*	*3*[b]	*0.21*	*0.099*	*3*[b]	*0.92*
Max		*3.6*	*0.232*	*0.67*[c]	*1.26*	*0.398*	*0.67*[c]	*1.41*
Variance		*2.4*	*0.013*		*0.36*	*0.03*		*0.06*

Significant *p*-values for F_{ST} are in bold font
Underlined F_{ST} values = 0 show negative F_{ST} values replaced with zeros
[a] the number of significant positive F_{ST} values
[b] the number of pairwise Fst values
[c] the proportion of significant positive pairwise F_{ST} values
