# Supplementary figures and images for: Does the Colonizing Population Exhibit a Reduced Genetic Diversity and Allele Surfing? A Case Study of the Midday Gerbil (Meriones meridianus Pallas) Expanding Its Range
Source: Animals (Basel). 2024 Sep 20;14(18):2720. doi: 10.3390/ani14182720 (PMC11429244; doi:10.3390/ani14182720)

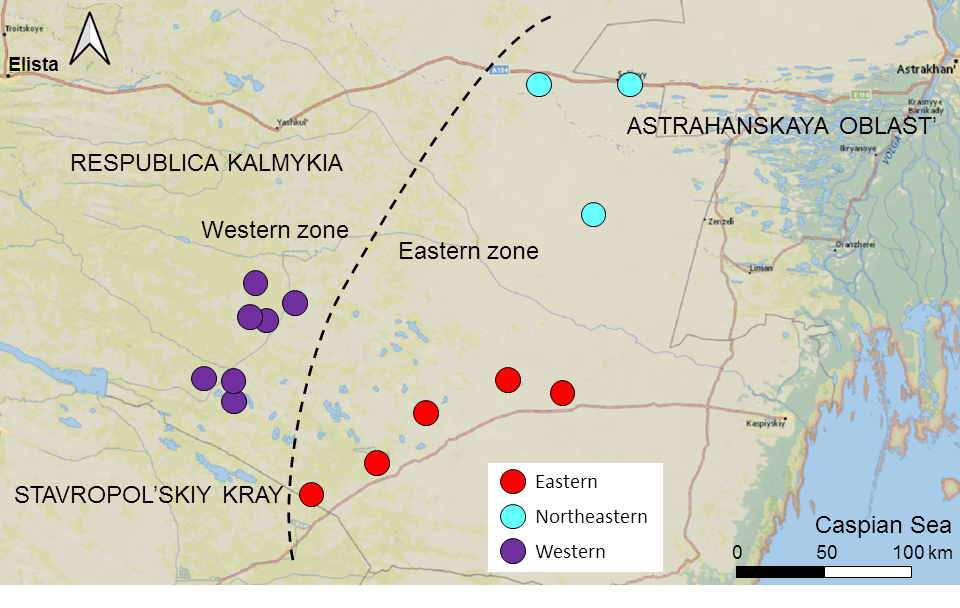

Supplement: Supplementary file 1 [file animals-14-02720-s001.zip › Figure_1.tif]

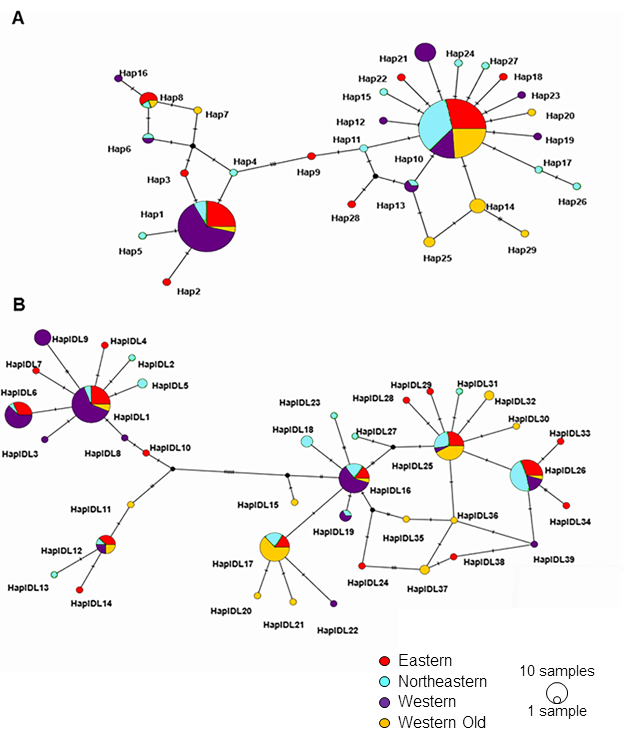

Supplement: Supplementary file 1 [file animals-14-02720-s001.zip › Figure_2.tif]

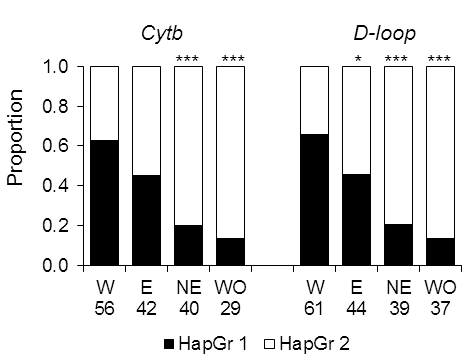

Supplement: Supplementary file 1 [file animals-14-02720-s001.zip › Figure_3.tif]

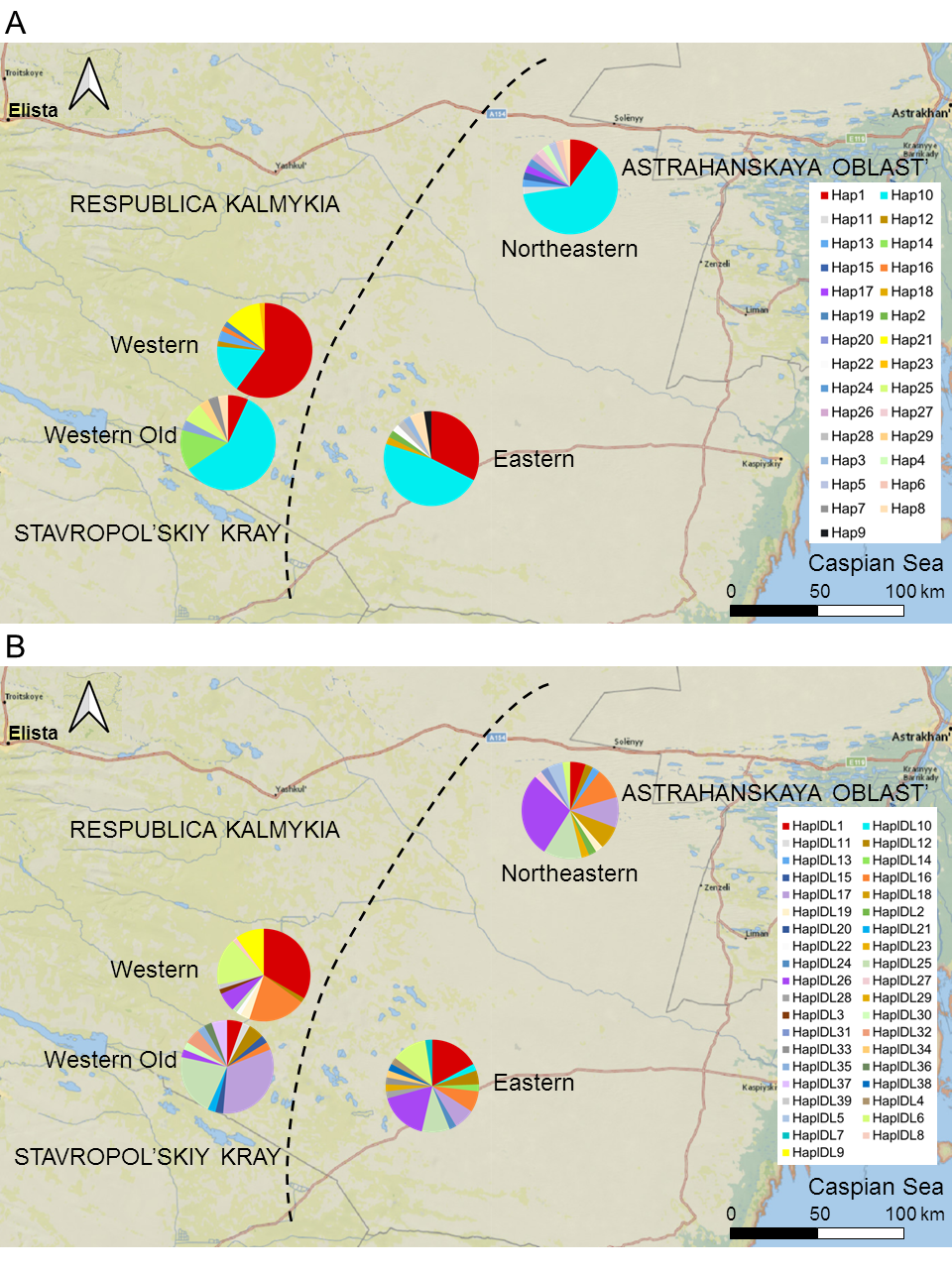

Supplement: Supplementary file 1 [file animals-14-02720-s001.zip › Figure_4.tif]

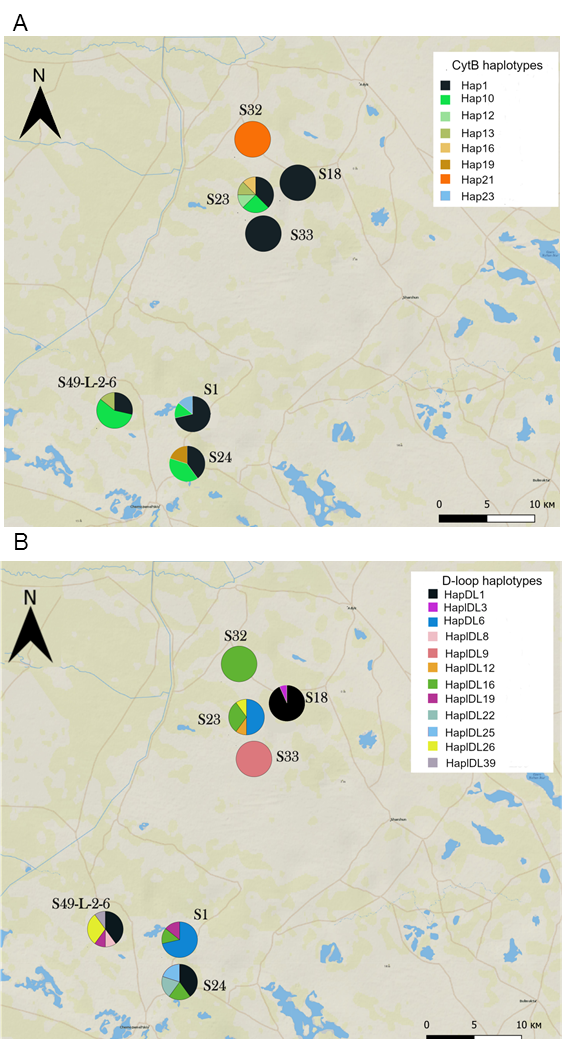

Supplement: Supplementary file 1 [file animals-14-02720-s001.zip › Figure_5.tif]

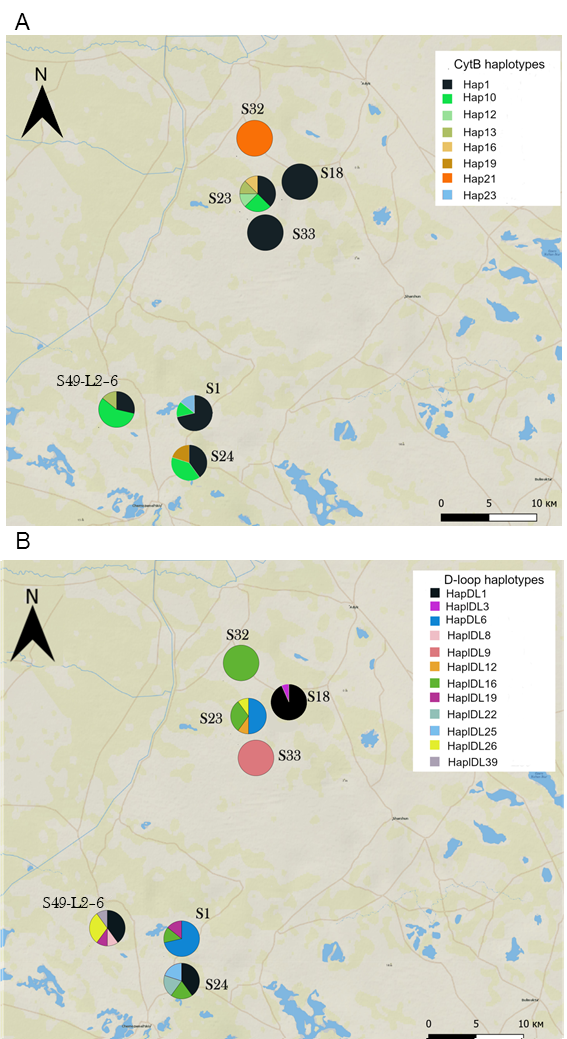

Supplement: Supplementary file 1 [file animals-14-02720-s001.zip › Figure_6.tif]
